# Autologous full-thickness skin graft as reinforcement in parastomal hernia repair: a randomised controlled trial

**DOI:** 10.1186/s13063-021-05884-4

**Published:** 2021-12-07

**Authors:** Viktor Holmdahl, Ulf Gunnarsson, Karin Strigård

**Affiliations:** 1 Department of Surgical and Perioperative Sciences, Surgery, Umeå University, Umeå, Sweden; 2 Sunderby Research Unit, Umeå University, Luleå, Sweden

**Keywords:** Parastomal Hernia, Full-thickness skin graft, IPOM, Open repair, Randomised controlled trial, Multicentre

## Abstract

**Background:**

Parastomal hernia is a common complication of an enterostomy and can have a significant impact on health-related quality of life. Currently used methods of repair have high recurrence rates and considerable risk for complications. We have developed a new technique for parastomal hernia repair that uses full-thickness skin graft as reinforcement.

**Methods:**

This study protocol describes a multicentre randomised controlled trial on parastomal hernia repair comparing a new full-thickness skin graft technique with conventional synthetic composite mesh as reinforcement of the abdominal wall. Patients with a symptomatic parastomal hernia will be included and followed up at 3, 12 and 36 months, with surgical complication as the primary outcome. Secondary outcomes will be recurrence rate and health-related quality of life assessed with VHPQ, EORTC C30 and CR29. Tissue biology and collagen metabolism will be investigated pre- and postoperatively using biopsies of the abdominal wall fascia and blood samples.

**Discussion:**

Parastomal hernia constitutes a major clinical problem where the prospects of a good result after hernia repair are presently poor. This new method of repair with full-thickness skin grafting could be a new alternative in our surgical toolbox, but before then, it must be evaluated properly.

**Trial registration:**

ClinicalTrials.gov NCT03667287. Registered on September 12, 2018

## Administrative information

Note: the numbers in curly brackets in this protocol refer to SPIRIT checklist item numbers. The order of the items has been modified to group similar items (see http://www.equator-network.org/reporting-guidelines/spirit-2013-statement-defining-standard-protocol-items-for-clinical-trials/).

**Table:** 

Title {1}	Stoma Hernia Intraperitoneal Full-Thickness Skin (SHIFT)
Trial registration {2a and 2b}.	Registered at clinicaltrials.gov; ID NCT03667287
Protocol version {3}	2021-05-07 Version 1.0
Funding {4}	Funded by grants from Visare Norr (VISARENORR939013), the Swedish Research Council (921-2014-7196) and a regional agreement between Umeå University and Västerbotten Region (ALF, nr VLL-545001). This investigation was also supported by grants from Lion's Cancer Research Foundation, Umeå University (LP 16-2142).
Author details {5a}	Viktor Holmdahl M.D. ^1, 2^ Ulf Gunnarsson M.D. PhD. ^1^ Karin Strigård M.D. PhD. ^1^ 1. Department of Surgical and Perioperative Sciences, Surgery, Umeå University 2. Sunderby Research Unit, Umeå University, Luleå, Sweden
Name and contact information for the trial sponsor {5b}	Trial Sponsor: Västerbotten Region, Surgical centre at the University hospital of Umeå Adress: Daniel Naezéns väg, 907 37 Umeå Contact name: Urban Arnelo Telephone: +4690 785 00 00 E-mail: urban.arnelo@regionvasterbotten.se
Role of sponsor {5c}	This funding source had no role in the design of this study and will not have any role during its execution, analyses, interpretation of the data, or decision to submit results. The sponsor is responsible for providing the necessary structural, economical and personnel resources to ensure the study participants safety and to carry out the study.

## Introduction

### Background and rationale {6a}

Parastomal hernia is the main complication of an enterostoma [1–3]. Occurrence rates up to 80% have been reported, with numbers varying considerably due to differences in diagnostic method, follow-up time, type of stoma, and the lack of a uniformly accepted definition of parastomal hernia [2–5]. Symptoms from a parastomal hernia range from asymptomatic to problems significantly impairing the patient’s quality of life, even life-threatening due to strangulation of the hernia content. There are few studies investigating the frequency of specific complaints, and there is thus little information clearly indicating which patients are likely to benefit from surgical repair [6].

Several techniques are presently used for parastomal hernia repair. The European Hernia Society recommends a synthetic mesh plasty, but scientific evidence on how and in what position the reinforcing material should be applied is weak. Even the best available methods today are associated with high recurrence rates and significant risk for complications that can be fatal [7–9]. The use of synthetic mesh material is associated with severe complications such as mesh infection, fistula formation and erosion of the intestines [9]. There are also patient associations and other communities that oppose the use of synthetic mesh material (https://www.facebook.com/groups/meshproblems/, https://www.facebook.com/MeshMeNot/, https://meshmenot.wordpress.com/, https://meshvictimsunited.org/).

Autologous full-thickness skin grafting (FTSG) has been shown to be a possible alternative to conventional synthetic mesh in hernia repair [10]. By avoiding the use of foreign materials, FTSG could offer safer and less expensive reinforcement in parastomal hernia repair. The technique of grafting autologous skin in hernia repair was successfully used before the advent of synthetic mesh, but its application in parastomal hernia is new [11, 12].

The present RCT follows a translational chain of research starting with a murine model which showed good FTSG survival in both intraperitoneal and onlay positions [13]. Furthermore, in an experimental model, fresh FTSG was shown to have equal or better tensile strength than both synthetic and biological reinforcement material, which is a prerequisite in hernia surgery [14].

The study intervention was developed with the aid of 3D-models reconstructed from computerised tomography (CT) scans of patients suffering from parastomal hernia. Four pilot patients have then been operated on in a feasibility study which showed no procedure-related complications [15].

### Objectives {7}

The objective of the trial is to compare a novel method of repair for parastomal hernia to one of the best available conventional methods in terms of surgical complications, recurrence, QoL and health economics.

Our hypothesis is that autologous FTSG as reinforcement material in parastomal hernia (PH) repair offers a safer and more comfortable alternative to conventional synthetic mesh material.

### Trial design {8}

The trial is a parallel group, superiority trial with an allocation ratio of 1:1.

## Methods: participants, interventions and outcomes

### Study setting {9}

This study will initially take place at three public hospitals in Sweden, one university hospital, one regional hospital and one county hospital, all situated in the north of Sweden. The university hospital is a tertiary referral centre for advanced abdominal wall reconstructive surgery.

### Eligibility criteria {10}

Consultant surgeons with long experience in abdominal wall surgery will be responsible for the assessment of potential study participants before inclusion and will attend all intervention procedures.

Inclusion criteria:

- Colo-, ileo- or urostomy
- Parastomal hernia diagnosed with intrastomal ultrasonography and/or CT-scan
- Symptoms from the parastomal hernia requiring surgical intervention
- 18 years of age
- Sufficient knowledge of the Swedish language assuring that questionnaires can be adequately understood and answered

Exclusion criteria:

- Cognitive impairment causing poor compliance to postoperative prescriptions and/or answering questionnaires
- Insufficient amount of good quality skin suitable for transplantation
- Expected high donor-site morbidity
- Fistula/e adjacent to stoma
- Mb Crohn
- Concomitant ventral hernia requiring mesh repair
- Other intra-abdominal disease requiring surgical intervention

### Who will obtain informed consent? {26a}

Information about the trial will be given to potential study participants per telephone or in person by the assessing surgeon or other responsible researcher. The potential candidate will then be assessed in the out-patient clinic by a surgeon regarding inclusion and exclusion criteria. If the potential study participant is eligible for inclusion, written and verbal informed consent will be obtained at the time of assessment after relevant questions have been answered. The potential study participant may also be given time for consideration and submit their consent at a later date.

### Additional consent provisions for collection and use of participant data and biological specimens {26b}

Patients included in the study will also be offered the opportunity to participate in an ancillary study investigating tissue biology of patients with parastomal hernia. Consent for participating in this ancillary study will be obtained in conjunction with acquisition of consent for the main study.

For more information, see the “Plans for collection, laboratory evaluation, and storage of biological specimens for genetic or molecular analyses for this trial/future use {33}” section.

## Interventions

### Reasons for the choice of comparators {6b}

There is weak evidence as to which method of repair is the best available at present. The European Hernia Society is against the use of suture repair as well as repair using a biological mesh [16]. In terms of recurrence, there is evidence favouring IPOM mesh using the Sugarbaker technique, but evidence is weak regarding morbidity.

In view of the fact that there is such weak evidence on how synthetic mesh should be applied to obtain the best results, our comparator was chosen for its similarity to the experimental intervention [15].

### Intervention description {11a}

The patients included in the study will be randomised to:

1. FTSG as reinforcement material. Surgery begins by marking the proposed area of skin above the planned laparotomy incision, while assuring that there will be enough skin left for primary skin closure. In case of insufficient skin appropriate for transplantation adjacent to the midline incision, other possible donor sites are the inside of the upper arm or thigh. The FTSG is excised sharply, dissected free from all subcutaneous tissue and knife-meshed with multiple small incisions (5–10 mm), plus a larger circular incision forming an orifice in the centre of the graft for the stomal intestine. Meshing serves to prevent seroma and haematoma formation, to facilitate ingrowth and to increase the area of the FTSG.

The FTSG is then placed in surgical gauze soaked in hydrogen peroxide awaiting implantation. A midline laparotomy is performed and adhesiolysis around the stoma is performed if necessary. The stoma is dissected free at the mucocutaneous junction, sealed with a temporary suture and retracted into the abdomen. The fascial defect is then reduced to appropriate size depending on the size of the patient’s stomal intestine, with interrupted single 2-0 PDS (Ethicon Inc. Cornelia, GA, USA) sutures. The stomal intestine is passed through an orifice created in the FTSG and sutured to the intestine with interrupted single 3-0 monocryl (Ethicon Inc. Cornelia, GA, USA) sutures with the epidermis facing the intra-abdominal contents.

The stoma is brought up through the reduced fascial defect and the FTSG is sutured to the peritoneum and abdominal wall under tension with interrupted 2-0 PDS sutures at intervals of 10–20 mm along the edges of the graft. Reinforcing sutures are also placed over the fascial reduction so that the forces in the abdominal wall are transferred from the fascial defect to the skin graft.

OR

2. Synthetic mesh as reinforcement. A midline laparotomy is performed and adhesiolysis around the stoma is performed if necessary. The stoma is dissected free at the mucocutaneous junction, sealed with a temporary suture and retracted into the abdomen. The fascial defect is then reduced to appropriate size depending on the size of the patient’s stomal intestine with interrupted single 2-0 PDS (Ethicon Inc. Cornelia, GA, USA). A DynaMesh®-IPST (FEG Textiltechnik mbH, Aachen, Germany) of appropriate size is then applied to the stomal intestine. The stoma is brought up through the reduced fascial defect, and the DynaMesh® is sutured to the peritoneum and abdominal wall with interrupted 2-0 PDS sutures at intervals of 10–20 mm along the edges of the graft. Reinforcing sutures are also placed over the fascial reduction so that the forces in the abdominal wall are transferred from the fascial reduction to the mesh.

### Criteria for discontinuing or modifying allocated interventions {11b}

Any study patient requesting to end their participation in the study will immediately be withdrawn from whatever stage they have reached in the study process without having to explain why.

The anatomy in the abdominal cavity as well as the current conditions at the time of surgery can prevent the feasibility of the planned procedure, in part or completely. Minor alterations to the surgical interventions may be accepted, such as isolated deviations from the specified intervals when suturing the reinforcement material to the abdominal wall. Major obstacles that make the planned procedure impossible to perform without significant risk, such as massive adhesions or other contraindications such as finding disseminated malignancy, will exclude the patient from further participation in the study.

### Strategies to improve adherence to interventions {11c}

It is a great challenge to standardise surgical procedures in detail, since difficulty increases with the complexity of the procedure and the target organ for the surgical intervention.

Thorough operation manuals with clarifying pictures have been prepared with step-by-step guidance of procedural details. When additional study centres are included, a surgeon responsible from one of the initially participating centres will assist at the first operations to further assure standardisation and procedural adherence.

Any inherent variability of the procedures remaining, such as different surgeons having different approaches for exposure and dissection, will increase the external validity of the study.

### Relevant concomitant care permitted or prohibited during the trial {11d}

No other intra-abdominal interventions are permitted concomitant to the planned intervention. Potential iatrogenic injuries which occur during the intervention should be managed according to surgical standards. Minor mid-line hernias which allow for suture repair can be managed during the intervention, but if requiring mesh reinforcement, the patient cannot be included in the study.

### Provisions for post-trial care {30}

Should any study participant suffer harm, he/she is entitled compensation under the Swedish Patient Injury Act. Since the study surgical intervention and all postoperative care is performed under the Swedish public healthcare system, all study participants will be covered by the Swedish regional health authorities mutual insurance company (Löf) [17].

## Outcomes {12}

### Participants will be followed up at 3, 12 and 36 months postoperatively.

#### Primary outcome:

- Rate of surgical complications at 3, 12 and 36 months. Complications will be assessed over a 3-year period at regular follow-ups by an independent surgeon who is unaware of which surgical method was used. Eventual complications include infection, bleeding, seroma, and fistula formation. Regarding recurrence, the best available methods of repair today are associated with complications that in some cases can be fatal [7].

#### Secondary outcomes:

- Recurrence. Parastomal hernia recurrence will be assessed clinically, with stomal ultrasound and/or CT-scan.
- Pain. All subjects will complete the Ventral Hernia Pain Questionnaire (VHPQ) to assess and compare preoperative pain ratings related to daily activities with ratings at all clinical follow-ups [18].
- Quality of life. All subjects will complete the European Organisation for Research and Treatment of Cancer questionnaire module for colorectal cancer (EORTC CR29) as well as the European Organisation for Research and Treatment of Cancer core quality of life questionnaire (EORTC QLQ-C30) to assess and compare different aspects of quality of life pre- and postoperatively at all clinical follow-ups [19–21].
- Health economics. Cost-effectiveness for each treatment arm. The total cost of each method will be calculated from the hospital healthcare costs system based on duration of surgery, cost of operating time and equipment, duration and cost of anaesthesia and cost of complications including infection, bleeding, seroma and fistula formation.

### Participant timeline {13}

The main timeline may be found in Table 1, and timeline for the ancillary study in Table 2.

**Table 1:** Time schedule SHIFT

	Study period				
	Enrolment	Operation	Months postoperatively		
Timepoint	− X*	0	***3***	***12***	***36***
**Enrolment:**					
**Eligibility screening**	X				
**Informed consent**	X				
**Allocation**		X			
**Interventions:**					
***Stomal ultrasound***	X			X	X
***CT-scan***	X**			X**	X**
**Assessments:**					
***Clinical characteristics***		X	X	X	X
***EORTC CR29/CR30***	X		X	X	X
***VHPQ***	X		X	X	X

CT, computerised tomography

*Time from enrolment to operation will vary

**When clinical evaluation and stomal ultrasound is insufficient

**Table 2:** Time schedule of ancillary study

	Study period					
	Preoperative	Operation	Postoperative			Analyses
Timepoint	***− 1 day***	0	***1 day***	***3 months***	***12 months***	***t***_***x***_
**Interventions:**						
***Fascia sample***		X				X
***Venous blood sample***	X	X	X	X	X	X
***Subgroup intervention:***						
***Needle biopsy***					X	X
**Assessments:**						
***List baseline variables***	X					
***List interventional variables***		X				

### Sample size {14}

The power calculation is based on the primary outcome with an estimated short-term complication rate of 15% in the FTSG-group and in 40% in the synthetic mesh group. To achieve 80% power and 95% significance level, 39 patients are required in each arm. To compensate for loss to follow-up over the relatively long follow-up time, we plan for 90 patients (45 in each arm) to be included in the trial.

### Recruitment {15}

The surgeons responsible for the decision to operate on a patient with parastomal hernia will provide information about the trial.

Furthermore, stomal dressing and basic stomal care is provided by stoma nurses who meet all stoma patients. Since stoma-related complaints are usually first pointed out to the stoma nurse by the patient, information about the trial will be presented to the stoma nurses at each participating hospital. In this manner, any potential candidates for inclusion will be referred by the nurses to the assessing surgeon. To increase the rate of inclusion, other study centres will be invited to participate in the trial. The trial is advertised at conferences, meetings and other forums in which representatives from other potential study centres are present.

## Assignment of interventions: allocation

### Sequence generation {16a}

The randomisation sequence is arranged in blocks of five with alternating overweight to either allocation obtained manually by research administrators. This eliminates the risk of skewing distribution at centres with low case volumes.

### Concealment mechanism {16b}

A total of 90 opaque and sealed envelopes numbered 1 to 90, each containing a paper with either the word “FTSG” or “Synthetic mesh,” 45 of each. They are kept locked-in at the research group’s office upon the day of randomisation.

### Implementation {16c}

The allocation sequence is generated by the research group administrators. Patients are enrolled by surgeons at the out-patient clinic and are provided a randomisation number correlating to a sealed envelope. The intervention arm is assigned after induction of anaesthesia when the accompanying randomisation envelope is opened.

## Assignment of interventions: blinding

### Who will be blinded {17a}

The allocated intervention is blinded to the patient, postoperative care providers and the surgeon performing the clinical follow-ups. The operating surgeons and the data analysts are not blinded.

### Procedure for unblinding if needed {17b}

If a patient suffers a complication that requires reoperation, the surgeon on call can be unblinded to the procedure that has been performed if it is deemed necessary to assess the indication for surgical intervention. Reoperation does not necessarily necessitate unblinding of the patient and the nursing staff nor does the trial follow-up surgeon need to be unblinded to the index procedure that has been performed.

## Data collection and management

### Plans for assessment and collection of outcome data {18a}

All clinical study data will be registered in an Access® database (Microsoft, Redmond, WA, USA).

- Baseline data are collected at the time of inclusion once assessment of eligibility by the including surgeon is complete. At this point, the surgeon is unaware of what intervention the patient will be allocated. Baseline data include:
  - BMI. Body mass index.
  - ASA (American Society of Anesthesiologists) class.
  - Current medical conditions
  - Current medication
  - Number and type of previous abdominal surgeries.
  - Type of and reason for stoma
- Operation data will be collected immediately after surgery by the surgeon. Data collected on completion of surgery:
  - Participating surgeons
  - Treatment allocation
  - Size of the hernia defect before and after reduction
  - Size of the FTSG
  - Length of stoma bowel above the fascia
  - Operating time
  - Minimum overlap of reinforcement material
- Data on postoperative care on the ward will be collected by the blinded nursing staff on a case report form (CRF). After discharge, the CRF will be collected and registered in the database. Parameters monitored daily during the entire time on the ward:
  - C-reactive protein
  - Serum albumin
  - Complete blood cell count
  - Serum creatinine
  - Blood glucose
  - Oxygen saturation
  - Experienced pain assessed on a visual analogue scale
- Clinical follow-up data will be collected by the blinded surgeon conducting the follow-up. This surgeon will be experienced in stoma surgery (i.e. colorectal, or abdominal wall surgery) but will not receive any specific training on the follow-up procedure in this study. Parameters that will be assessed at the clinical follow-up:
  - BMI
  - Clinically judged recurrence
  - Surgical complication (seroma, infection, other)
  - Stoma complication (stenosis, necrosis, other)
  - Readmission or reoperation during the follow-up period
  - Pain assessed on a visual analogue scale
  - Experienced improvement assessed on a visual analogue scale
  - Abnormal healing process or poor aesthetic result of the operation scar
  - Compliance to the postoperative girdle prescribed
- Questionnaires both preoperative and during the follow-up period will be collected by the research group administrators and stored in a separate Access database. Questionnaires used in the study are:
  - VHPQ, which is a validated questionnaire specially designed to evaluate pain in relation to daily activities following ventral hernia repair. VHPQ was developed and validated by our research group and focuses on pain behaviour [18].
  - EORTC C30 and CR29, questionnaires developed by the European Organisation of Research and the Treatment of Cancer, are used to explore and quantify different aspects of health-related quality of life (QoL) in patients with cancer. C30 constitutes the “core” questionnaire with a broad range of questions including social, emotional and physical aspects of cancer [20, 21]. CR29 focuses on symptoms specific for colorectal cancer and includes a section on stoma-specific problems [19]. These questionnaires are validated and shall be answered together. The questionnaires are interpreted with designated scoring manuals.

### Plans to promote participant retention and complete follow-up {18b}

Patients will be sent an appointment for clinical follow-up by mail. Administration of patient follow-up will be conducted by a research nurse with overview of the entire study population (checklists). All data that has been collected up to the point of discontinuation will be considered consensual and used in the analyses. After discontinuation no further data will be collected from the study participant which will be considered “lost to follow-up”.

### Data management {19}

Data will be collected on case report forms (CRF) for each step of the study (inclusion, intervention, postoperative care, and each follow-up separately). The forms will be coded with the individual randomisation number and stored in locked cabinets with logged access only available to the researchers and administrators responsible for the trial. When data collection is complete, the CRF will be transferred to an Access® (Microsoft®, Redmond, WA, USA) database, stored in encrypted form on the intranet of Region Västerbotten, with the password only available to the responsible researchers and administrators. Microsoft Access® is specially designed for databases and includes rules and limitations for data input and analyzation, which prevents duplicates and significantly decreases the risk for data corruption. CRFs are checked by an experienced research administrator before entering data to the database. The Access® database is designed so that all parameters included are limited to realistic values to promote data quality.

### Confidentiality {27}

Patients that are potential candidates for inclusion are cared for under the national healthcare system for their stoma. As soon as a patient, stoma nurse, or any other healthcare provider notices a stoma problem that could be a parastomal hernia, that patient will be referred to one of the surgeons responsible for handling of patients in the study. This procedure is the same as for patients not taking part in the study, and all information on the management of potential study candidates is stored in the medical records as is normal clinical practice. For more information on how confidentiality is assured, please see the “Data management {19}”.

### Plans for collection, laboratory evaluation and storage of biological specimens for genetic or molecular analyses for this trial/future use {33}

Patients who participate in the ancillary study investigating tissue biology in parastomal hernia repair undergo biopsy of the abdominal wall fascia during the primary intervention. This biopsy is analysed for collagen structure and metabolism, tissue composition and protein expression. Postoperatively and during the clinical follow-up, blood samples are taken to analyse markers monitoring connective tissue turnover and tissue remodelling processes.

A subgroup of patients will also be offered postoperative biopsy of the implanted reinforcement material via a transcutaneous ultrasound-guided needle biopsy adjacent to the stoma.

All tissue samples will be stored fresh-frozen in ultra-low temperature freezers (at Biobanken Norr) until analysis.

## Statistical methods

### Statistical methods for primary and secondary outcomes {20a}

Generally, statistical methods used in the study are aimed to describe and compare the outcomes between the study groups. Based on the characteristics of each outcome, chi-squared test, Mann-Whitney *U* test and Student’s *t* test are used to evaluate the statistical significance of the different outcomes.

#### Rate of complications and recurrences

Data are presented descriptively and the results from each study group are compared with chi-squared test.

#### Pain and quality of life

Evaluated with VHPQ and EORTC CR29 and CR30 respectively and answers are reported and scored according to their specific scoring manuals. Comparisons between the study groups are done with Mann-Whitney *U* test or Student’s *t* test by convention.

#### Health economics

Data are presented descriptively and the results from each study group are analysed with Mann-Whitney *U* test or Student’s *t* test depending on the distribution of the specific variable.

The study assumes a significance level of 0.05.

### Interim analyses {21b}

After 30 operations have been performed, a safety analysis is performed. It is conducted by a scientifically experienced researcher (at least assistant professor) with long clinical experience of abdominal wall surgery but has no connection with the study. The safety analysis is based on CRF data and complications graded Clavien-Dindo 3b or worse based on information from copies of the medical records from postoperative care on the ward and clinical follow-ups. Should serious or unexpected complications associated with study allocation arise, the study will be interrupted by the surgeon performing the safety analysis. Safety analysis data will otherwise not be available to the responsible researchers or surgeons participating in the study.

### Methods for additional analyses (e.g. subgroup analyses) {20b}

Regression analysis is used to explore any relationships between patient characteristics and outcome. Depending on the characteristics of the outcome, different concepts for regression analyses are used. Linear regression is used for continuous variables, logistic regression for dichotomous variables and ordinal regression for ordinal outcome variables.

### Statistical methods to handle protocol non-adherence and missing data {20c}

Study outcomes are analysed as per protocol. Patients are randomised in the operating theatre after induction of general anaesthesia and the only scenario where the patient does not receive the intervention allocated is if it is deemed technically impossible after the laparotomy has been performed or if the patient suffers an anaesthetic complication before the randomisation envelope is opened. In both these scenarios, the patient cannot be operated with any intervention so chance should evenly distribute this specific form of non-adherence.

No statistical methods are used to compensate for missing data or loss to follow-up. The power calculation is dimensioned to enable enough statistical power with up to 12 missing patients in all.

### Plans to give access to the full protocol, participant level data and statistics code {31c}

Details of the full protocol, participant-level dataset, or the statistics code are not available to the public. Data not published may be made available upon reasonable request to the researchers responsible or corresponding authors of the publications.

## Oversight and monitoring

### Composition of the coordinating centre and trial steering committee {5d}

The university hospital serves as an administrative hub for the trial, hosting a central office with administrative staff. This central office coordinates the trial, monitors the trial progress and can aid all included study centres with administrative support during office hours. The trial is governed by a steering committee consisting of four researchers clinically active on the three initial study centres and an administrator. They will provide informational mailings to the responsible surgeon and administrative staff on all study centres twice a year and invite to an annual physical meeting.

### Composition of the data monitoring committee, its role and reporting structure {21a}

A safety analysis will be performed by an independent senior surgeon after 30 operations (more details under item 21b). This person will be certified to terminate inclusion in the study if undefendable differences between the treatment arms are found or if repeated method related severe adverse events are found in any of the study arms. If no such signs are found, results from the safety analysis will not be propagated to the steering board or participating centres. No other progressive data monitoring will be performed in this trial.

### Reporting adverse events and harm {22}

Adverse events and surgical complications are the main outcome of the trial and will be documented in the medical records. If they occur, they will be included in the CRF.

### Frequency and plans for auditing trial conduct {23}

Other than the safety analysis, no other auditing is planned.

### Plans for communicating important protocol amendments to relevant parties (e.g. trial participants, ethics committees) {25}

Any protocol modification deemed necessary will first be reviewed by the Swedish Ethics Review Authority and then registered at ClinicalTrials.gov. This information will also be shared personally with the researcher responsible at each study centre.

### Dissemination plans {31a}

The results of the trial will be published in peer-reviewed open-access journals and presented at both national and international conferences.

## Discussion

Despite the relative frequency of parastomal hernia, there is a considerable lack of knowledge regarding epidemiology, symptomatology, indication for surgery and best practice when surgically managing the condition [1–3, 6]. The European Hernia Society guidelines on prevention and treatment of parastomal hernia provide predominantly weak evidence for both the epidemiological aspects of the disease and its subsequent treatment [16]. Since there is a general lack of studies comparing different techniques for parastomal hernia repair, there is no consensus on what the best method available is. This makes the design of a trial, and in particular, choosing a comparator to evaluate a novel treatment, difficult and even controversial. Our choice of using reinforcement with DynaMesh®-IPST as a comparator was made because of its similarity to our FTSG-method and with the background of recurrence rates in the vicinity of other IPOM methods [22]. Furthermore, recurrence of a parastomal hernia after surgical repair is of minor importance compared to the serious and sometimes fatal complications of repair methods using synthetic mesh as reinforcement. The primary goal of benign surgery must be the safety of the patients, which the primary outcome of this trial.

Both methods investigated in this trial are open techniques despite the increasing number of colorectal interventions being performed laparoscopically. This is primarily because it is not possible to apply an FTSG to the abdominal wall using conventional laparoscopic tacks. Therefore, to avoid cyst formation, an open technique with better control over the sutures was considered safer for the patients given the importance of the FTSG application under tension.

An advantage of using FTSG is that this reinforcement material is usually readily available in the quantity required. FTSG has been shown to be a safe and potentially useful alternative in giant incisional hernia repair [10, 23].

## Trial status

Current recruitment. The first patient was recruited December 2019. The global COVID-19 pandemic has unfortunately reduced resources available for major benign surgery and thereby prevented inclusion. Due to uncertainty about how the pandemic will affect the healthcare system in Sweden in the future, we cannot say when recruitment is likely to be completed. Our aim is to complete recruitment by December 31, 2025.

## Abbreviations

FTSG: Full-thickness skin graft
RCT: Randomised controlled trial
CRF: Case report form
VHPQ: Ventral hernia pain questionnaire
EORTC: European Organisation of Research and Treatment of Cancer
BMI: Body mass index
ASA: American Society of Anesthesiologists
IPOM: Intraperitoneal onlay mesh
QoL: Quality of life
